# Supplementary material for: Resolving electron and hole transport properties in semiconductor materials by constant light-induced magneto transport
Source: Nat Commun. 2024 Jan 5;15:316. doi: 10.1038/s41467-023-44418-1 (PMC10770130; doi:10.1038/s41467-023-44418-1)
Supplement: Supplementary file 1 — Supplementary Information [file 41467_2023_44418_MOESM1_ESM.pdf]

Resolving electron and hole transport properties in semiconductor materials by constant light-induced magneto transport

Supplementary information

1. CLIMAT module and method description

1.1 (100) CLIMAT measurements module design and overview

The design of the setup module (100) used in constant light-induced magneto transport (CLIMAT) measurements is shown in **Figure S1**. The module is designed to probe semiconductor, semi-insulating, and insulation materials. The current source 1 (205 or 304 for DC magnetic field method), current source 2 (208 and 308), current meter (206 or 305), and source meter (207 and 306), which control currents and probe Hall and conductivity voltages, are connected using connections line (101) with Hall effect insert card (103), which supports studied sample (107) and LED (109). LED (109) provides continuous illumination and is mounted to the Hall effect card by using an LED holder (108). To induce the Hall effect voltage, the magnetic field is generated using a magnet (104). The magnet field value is measured by using a Gauss probe (106). As a magnet field supply, various sources can be used: electromagnet, permanent magnet or other magnetic field sources. The CLIMAT can be measured using both an alternating magnetic field (AC) and a constant magnetic field (DC).

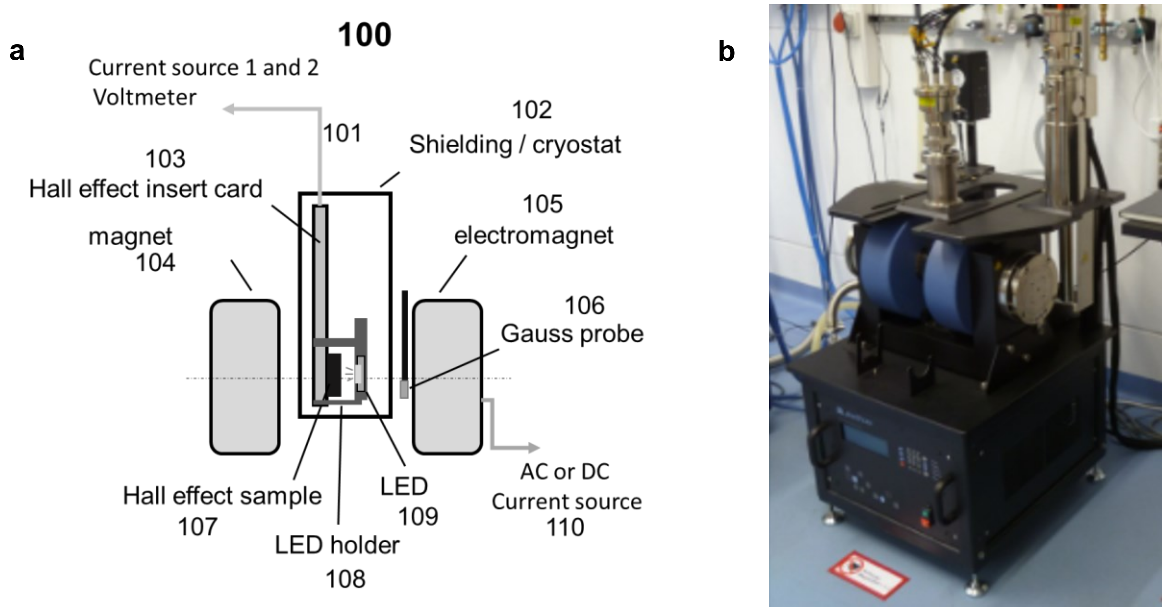

Figure S1 | CLIMAT Photo-Hall Effect measurements module design (a) Schematic drawing of CLIMAT module design; (b) image of the experimental setup.

The method utilizing AC magnetic field (Method A) is described separately in instrument part (200) and program part (600), which controls measurements. The DC measurements (Method B) are also described separately in instrument part (300) and measurement program part (700). To calculate the carrier's transport properties, programs (700 and 600) use algorithm 1 (400) for mixed electron and hole signals or algorithm 2 (500) for single carrier signals.

Two correction algorithms are developed for the case of parasitic conductivity (correction algorithm 1 (900)) and for the case of low Hall signals (correction algorithm 2 (1000)). The implementation and test of the CLIMAT method are given for p-type silicon with much higher electron than hole mobility (800 and **Figure 2**) and for p-type perovskite material with similar electron and hole mobility (1100 and **Figure 4**). The correction algorithms 1 (900) and 2 (1000) are implemented for the perovskite sample test (1100), which show a low signal at high intensity due to the compensation of free holes by electrons. Further, we discuss each step of the method in detail. The *p*-type materials are presented as implementation and test examples, but the same CLIMAT equations, module design, calculation algorithm, correction algorithm, and program are also valid for n-type material.

Table S1. Comparison of methods resolving charge transport properties of free carriers based on Hall measurements where σ, R_H , and μ_H are conductivity, Hall coefficient, and Hall mobility.

Method	Mobility	Concentration	Charge transpor properties	Parasitic conductivity detection and correction
Hall (1849)[3]	μ_H	$n = \frac{1}{e(R_H)}$	x	x
Hoshl *et al*[6] 1978	x	n≠p<br>calculated numerically	x	x
Rozenshweig et al.[7] 1982	x	n≠p<br>calculated numerically	x	x
Chen and Podzorov et al.[11] 2016	$\mu_h \gg \mu_e$	$n = \frac{1}{e(R_H)}$	$\tau, L,$*<br>D_e, D_h	x
Musiienko et al.[9] 2018	μ_h	n≠p<br>calculated numerically	x	x
Gunawan et al. [8] 2019	$\mu_h = \mu_{H\,dark}$<br>$\Delta\mu \;\; = (2 + \frac{dlnRh}{dln\sigma})\sigma Rh$	$\Delta n = \Delta p == \frac{\sigma(1 - \mu_e/\mu_h) - e\Delta\mu p_0}{(e\Delta\mu(1 + \mu_e/\mu_h))}$<br>$\frac{\mu_e}{\mu_h} = \frac{2\sigma(\Delta\mu - \sigma R_H) - e\Delta\mu^2 p_0 \pm \Delta\mu\sqrt{ep_0} \cdot}{(2\sigma(\Delta\mu - \sigma R_H))} \underline{\cdot \sqrt{e\Delta\mu^2 p_0 + 4\sigma(\sigma R_H - \Delta\mu)}}$	$\tau, L,$*<br>D_e, D_h	x
Bruevich and Podzorov et al.[44] 2021	$\Delta\mu_e = (\mu_h - \mu_{photo-Hall})\frac{\sigma}{\sigma - \sigma_0}$	$\Delta n = \Delta p = \frac{\sigma - \sigma_0}{(e(\mu_h + \mu_e))}$	$\tau, L,$*<br>D_e, D_h	x
This study 2023	$\begin{cases} p \gg n \Rightarrow \mu_h = \mu_H; \\ n \gg p \Rightarrow \mu_e = \mu_H; \\ p = n \Rightarrow \mu_H = \lvert \mu_h - \mu_e \rvert = \Delta\mu. \end{cases}$<br>$\begin{cases} p \gg n \wedge \mu_h > \mu_e \Rightarrow \mu_e = \mu_h - \Delta\mu \\ p \gg n \wedge \mu_h < \mu_e \Rightarrow \mu_e = \mu_h + \Delta\mu \\ n \gg p \wedge \mu_e > \mu_h \Rightarrow \mu_h = \mu_e - \Delta\mu \\ n \gg p \wedge \mu_e < \mu_h \Rightarrow \mu_h = \mu_e - \Delta\mu \end{cases}$	Both electron and hole properties<br>$n = \frac{\sigma \times (\mu_h - R_H\sigma)}{(e(\mu_h\mu_e + \mu_e\mu_e))}$<br>$p = \frac{(^{\sigma}/_{e} - n \cdot \mu_e)}{\mu_h}$	$\tau_e, \tau_h,$<br>L_e, L_h<br>D_e, D_h<br>$QFLS_e$<br>$QFLS_h$<br>η	$\mu_h - \mu_e = \frac{(\mu_{H2}\sigma_2 - \mu_{H1}\sigma_1)}{(\sigma_2 - \sigma_1)}$<br>$\sigma_S = \sigma_0 - e\mu_h p_0$<br>$p_0 = \mu_H \sigma_0/(q_e(\mu_h)^2)$

X – incapable; *Property of only one carrier type

Table S2. Comparison of methods resolving charge transport properties of free carriers: Space Charge Limited Current (SCLC), Drive-level Capacitance Profiling (DLCP), Impedance Spectroscopy (IS), Time-resolved Photoluminescence (trPL), Time-Resolved Microwave Conductivity, Optical-Pump Terahertz-Probe (OPTP), Photoluminescence (PL), carrier-resolved photo-Hall (CRPH), Time of Flight (ToF), and the Constant Light Induced Magneto Transport (CLIMAT).

Method	Steady-state properties	Resolves concentration of holes and electrons	Resolve transport properties of holes and electrons	Number of properties	Charge transport properties
DLCP[63,64]	☹	☹	☹	1	$N_t(x)$
trPL[1]	☹	☹	☹	1	τ^*
SCLC[64–67]	☹	☹	☹	2	N_t, μ^*
IS[68]	☹	☹	☹	2	μ, n or p^*
Hall effect[3]	☹	☹	☹	2	μ_H, n or p
PL[69,70]	☺	☹	☹	2	τ, QFLS*
Photoconductivity[11]	☺	☹	☹	3	n+p, $\mu_h+\mu_e$ τ^*
TRMC[1]	☹	☹	☹	4	n+p, $\mu_h+\mu_e$ τ^*, L^*
TRTS/OPTP[1]	☹	☹	☹	4	n+p, $\mu_h+\mu_e$ τ^*, L^*
ToF[71,72]	☹	☺ /☹- only low injection regime, transient	☺ /☹- only low injection regime, transient	4	μ_h, μ_e τ_e, τ_h
CRPH[8]	☺	☹	☹	7	Δn= Δp μ_h, μ_e τ^*, L^*, D_e, D_h
Photo-Hall[44]	☺	☹	☹	7	Δn= Δp μ_h, μ_e τ^*, L^*, D_e, D_h
**CLIMAT This study**	☺	☺	☺	14	n, p μ_e, μ_h, τ_e, τ_h, L_e, L_h D_e, D_h $QFLS_e$,$QFLS_h$ QFLS, η

☺ -- capable; ☹- incapable; *Property of only one carrier type

1.2 (200) CLIMAT Photo-Hall effect measurements set-up instrument's part with the alternating magnetic field (Method A)

To use the CLIMAT method, the instruments and their interconnections shown in **Figure S2** are needed. The magnet (201) is driven by AC current source (203) and controlled by the program (211). The magnetic field frequency and amplitude are controlled by the Gauss probe (209), which is additionally connected to Lock in amplifier (204) as a reference signal.

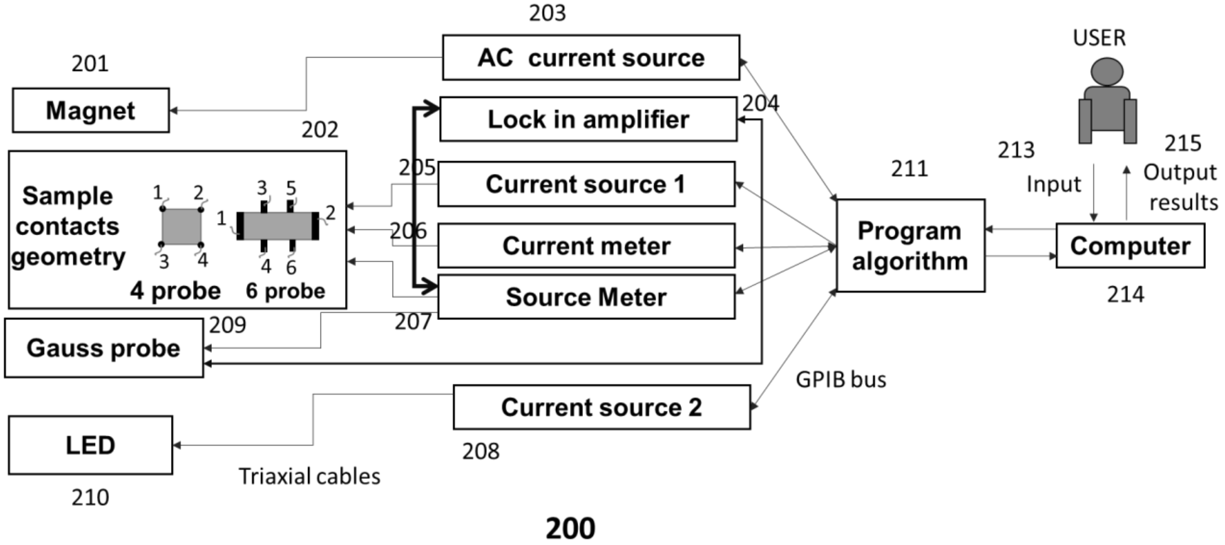

Figure S2 | CLIMAT measurements set-up instruments part with alternating magnetic field (Method A).

The studied sample is loaded using 4-probe or 6-probe contact geometry card (202) electrically connected with the current source (205), current meter (206), and source meter (207). Current source 1 (205) supplies electrical current through the sample measured by a current meter. Source meter (207) probes the voltage V for conductivity calculation and voltage V_H for Hall effect coefficient calculation (measured on the Lock in amplifier output).

In addition to dark Hall measurement, the measurements with light are performed to unlock charge transport properties of holes and electrons. Then, the sample is continuously illuminated by LED (210). The light power is controlled by the current source 2 (208). All instruments are driven by the program algorithms (211) installed on the computer (214), which is in turn controlled by the operator' input (213) where the operator can set up LED power, amplitude and frequency of magnetic field, the timeframe of the measurements, current through the sample, lock in amplification parameters, and a number of measured points (N).

In 4-probe geometry (see block 202), conductivity voltage is measured through the contacts 1-2, 2-4, 3-4, 1-3 when the current is applied to 3-4, 1-3, 1-2, 2-4 contacts. In the 6-probe contact geometry, the current is applied by contact 1-2, and conductivity voltage is probed by 3-5 and 4-6. For the Hall effect voltage, diagonal contacts are used to apply the current 1-4 and 2-3, and opposite diagonal contacts 2-3 and 1-4 are used to probe Hall voltage, respectively. Finally, contacts 3-4 and 5-6 were used to probe the Hall voltage when 6-probe 1-2 contacts were used to supply the current through the sample.

The voltage must be probed with both negative and positive currents and negative and positive magnetic fields to prevent the influence of sample non-uniformity. The values measured with negative and positive currents or magnetic field must be averaged, and the next voltages probed on the different contacts must also be averaged at the same condition (for example, $V = (0.5(V_{12I+} - V_{12I-}) + 0.5(V_{24I+} - V_{24I-}) + 0.5(V_{34I+} - V_{34I-}) + 0.5(V_{13I+} - V_{13I-}))/4$). In the case of considerable voltage offset on the contacts, the voltage offset measured at a zero current must be subtracted $\vec{V} = \vec{V_I} - \overrightarrow{V_{I=0}}$ and $\overrightarrow{V_H} = \overrightarrow{V_{H\_I}} - \overrightarrow{V_{H\_I=0}}$ to neglect the influence of the offset on the data.

As an output (215) the user receives twelve material properties– n, p, μ_e, μ_h, τ_e, τ_h, L_e L_h, D_e, D_h, QFLS, η –as a function of illumination intensity or carrier density. Additional raw data of conductivity, Hall coefficient, Hall mobility, parasitic conductivity and dark carrier concentration can be demonstrated.

Case of intrinsic semiconductor. In the case of an intrinsic material, the intrinsic carrier concentration (n_{int}) can be determined using the following relation: $n_{int} = \sqrt{N_c N_V} exp(-Eg/(2k_bT))$. Here, E_g represents the bandgap energy, k_b is the Boltzmann constant, T is the temperature, and Nc and Nv are the density of states in the conduction and valence bands, respectively. Nc and Nv can be derived from the effective electron (m_e) and hole (m_h) masses by $N_c = 2(\frac{2\pi\, m_e k_b T}{\hbar^2})^{1.5}$ and $N_v = 2(\frac{2\pi\, m_h k_b T}{\hbar^2})^{1.5}$.

The concentration of carriers in the dark and the drift mobility can be determined by incorporating an additional equation for the intrinsic carrier density ($n_0 = n_{int}^2/p_0$) into Eq. 3:

$$\mu_{H,0} - \frac{e}{\sigma_0}\left(\left(\frac{\left(\frac{\sigma_0}{e} - \Delta\mu\frac{n_{int}^2}{p_0}\right)}{\left(p_0 + \frac{n_{int}^2}{p_0}\right)}\right)^2 \cdot p_0 - \left(\frac{\left(\frac{\sigma_0}{e} - \Delta\mu\frac{n_{int}^2}{p_0}\right)}{\left(p_0 + \frac{n_{int}^2}{p_0}\right)} + \Delta\mu\right)^2 \frac{n_{int}^2}{p_0}\right) = 0 \qquad \text{S1}$$

$$\mu_h = \frac{\left(\frac{\sigma_0}{e} - \Delta\mu\frac{n_{int}^2}{p_0}\right)}{\left(p_0 + \frac{n_{int}^2}{p_0}\right)} \qquad \text{S2}$$

Here, p_0 can be determined from Eq. S1, and n_0 can be found as $n_0 = n_{int}^2/p_0$. The major carrier mobility can then be calculated using Eq. S2. Additionally, the minor carrier mobility can be determined using the CLIMAT approach presented in the main text: $\mu_e = \mu_h + \Delta\mu$.

The generation rate is directly found ($G = P \times \lambda \times 10^{-9}/(h \times c \times 10^3 \times d)$) by using calibrated photo-detector, where λ, c, and h are light wavelength, speed of the light, and plank constant.

1.3 CLIMAT measurements set-up instrument's part with the constant magnetic field (Method B)

The DC measurements of CLIMAT are performed with similar instruments as (200). With DC setup Lock in amplifier is not used, and the Hall voltage is directly probed from contacts (303) by the source meter (306).

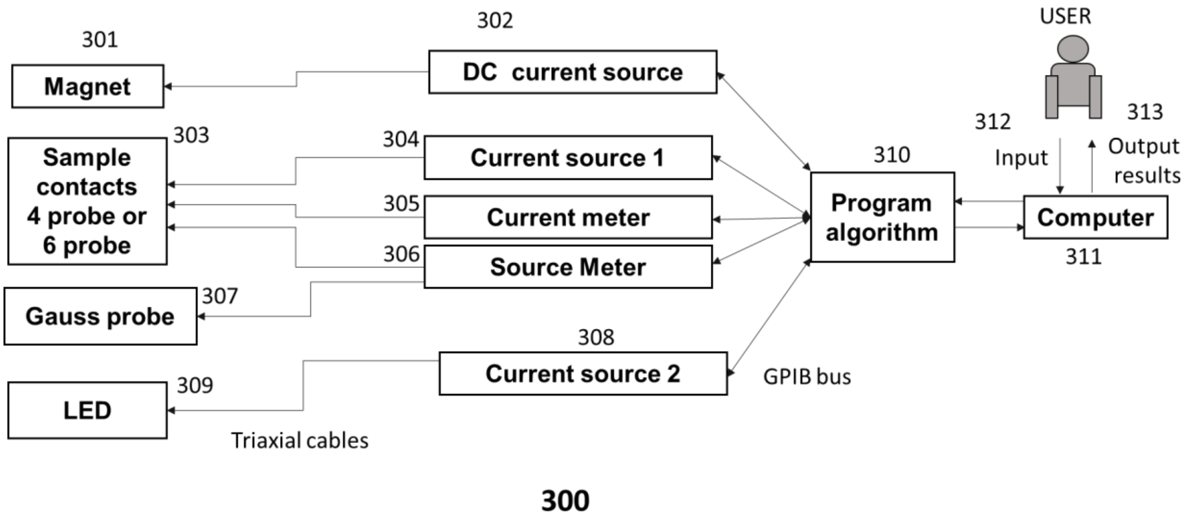

Figure S3 | CLIMAT measurements set-up instruments part with the constant magnetic field (Method B)

1.4 Algorithm 1: CLIMAT method for mixed electron and hole signals

The main algorithm implemented CLIMAT method is demonstrated in **Figure S4**. The measurement starts with the module (100) and instruments (200 or 300). The first step is the LED calibration with a light power meter (401) and calculation of the generation rate using (401) where $h = 6.6 \times 10^{-34}$ J·Hz - plank constant, c= 3×10^{8} m/s- speed of the light, $d = 3 \times 10^{-7}$ m - sample thickness, $\lambda = 670$ nm . Next, the conductivity voltage and Hall voltage are measured as a function of the illumination generation rate to find the Hall coefficient ($R_H = V_H \cdot d/(I \cdot B)$), conductivity ($\sigma = I \cdot d\ /(V\ S)$), and Hall mobility ($\mu_H = \sigma\ R_H$) a function of illumination intensity (402).

In this study, we do not observe the effect of the ion migration on the measurements due to the very low ion mobility and corresponding low to the electrical field of 10 V cm^{-1} used in the measurements. As a results the long transit time of mobile ions which reaches 10000 s [10,73] in samples with contact distance of 2 mm which is much larger that typical measurement tyme of 100 s. To prevent sample degradation on ambient air, samples were encapsulated and stored in a dry glovebox. We measured sample resistivity to control possible sample degradation. We observed constant resistivity of 2×10^{8} Ω cm before and after all electrical measurements, confirming the negligible effect of illumination or bias on sample degradation.

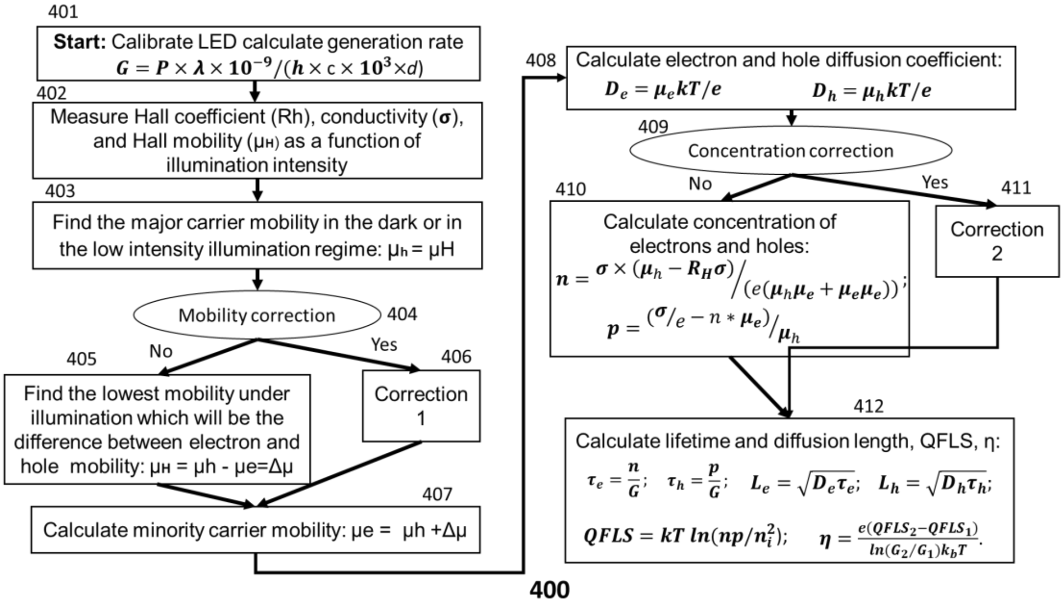

Figure S4 | CLIMAT method for mixed electron and hole signals (Algorithm 1)

The analysis of Hall mobility as a function of generation rate is used to find electron and hole mobility. The major carrier mobility is found in the dark ($\mu_h = \mu_H$) or in the low-intensity illumination regime (*p*>>*n*) if the sample is too resistive to show a signal in the dark (according to Equations 3-4). The difference between electron and hole mobility $\mu_H = |\mu_h - \mu_e|$ is then found at high intensities where concentration of holes and electrons merge (405); at such conditions, Hall mobility shows saturation (see **Figure S10** and **Figure 2**). By knowing major carrier mobility (hole) and the difference between hole and electron mobility, minor carrier mobility (electron) can be found (407).

In the case of parasitic conductivity or/and low Hall signal or/and high doping of the sample ($N_D > 10^{15}$ cm^{-3}), the correction step (406) using correction algorithm 1 (901) is used to calculate μ_h and μ_h. The parasitic conductivity can include surface conductivity, ionic conductivity, grain boundaries conductivity, and conductivity non-uniformities influence[45,74].

The next steps include the calculation of electron and hole diffusion coefficient (408) using known values of electron and hole mobility. The concentration of holes and electrons is calculated (410) using Equations 1-2. In the case of a low Hall signal (if μ_h and μ_e are similar), the correction step (409) and (411) is performed to find carrier concentration using an algorithm (1000). By knowing concentration (*n* and *p*), mobility (μ_h and μ_e) and diffusion coefficient (D_h and D_e) the charge transport parameters – lifetime, and diffusion length, can be found for electrons and holes separately (412). Quasi Fermi level splitting (QFLS) and ideality factor (η) can be further calculated (412) to predict the performance of the studied semiconductor as an active material.

1.5 Algorithm 2: Photo-Hall effect method for single carrier signals

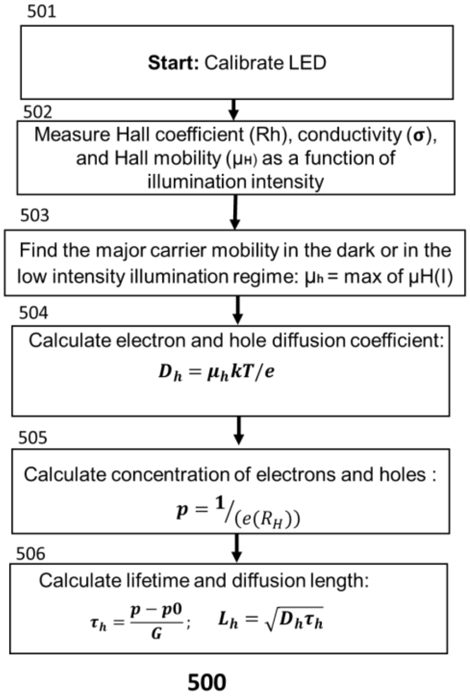

Figure S5 | CLIMAT method for single carrier signals

One carrier algorithm **Figure S5** (500) can be used if the samples which show a negligible minority carrier signal (for example, typical electron transport or hole transport materials used in p-i-n solar cells). Similar steps are performed to calibrate LED (501), and find conductivity, Hall coefficient and Hall mobility as a function of generation rate (502). Next majority carier mobility (503), diffusion coefficient (504), concentration (505) and transport parameters (506) are found. In step (503) the mobility correction algorithm 1 (900) can be used in the case of parasitic conductivity. Also, correction algorithm 2 (1000) can be used in step (505) to correct the concentration using conductivity valuers in the case of an undetectable Hall effect signal.

1.6 CLIMAT measurements program

The program (600) controlling module (100), instrument (200), and utilizing data processing algorithms (400) and (500) are demonstrated in **Figure S6**. Program Steps (601-611) demonstrate the necessary operations with the instrument, module and data to implement CLIMAT for a material sample study in AC magnetic field regime Method A (200).

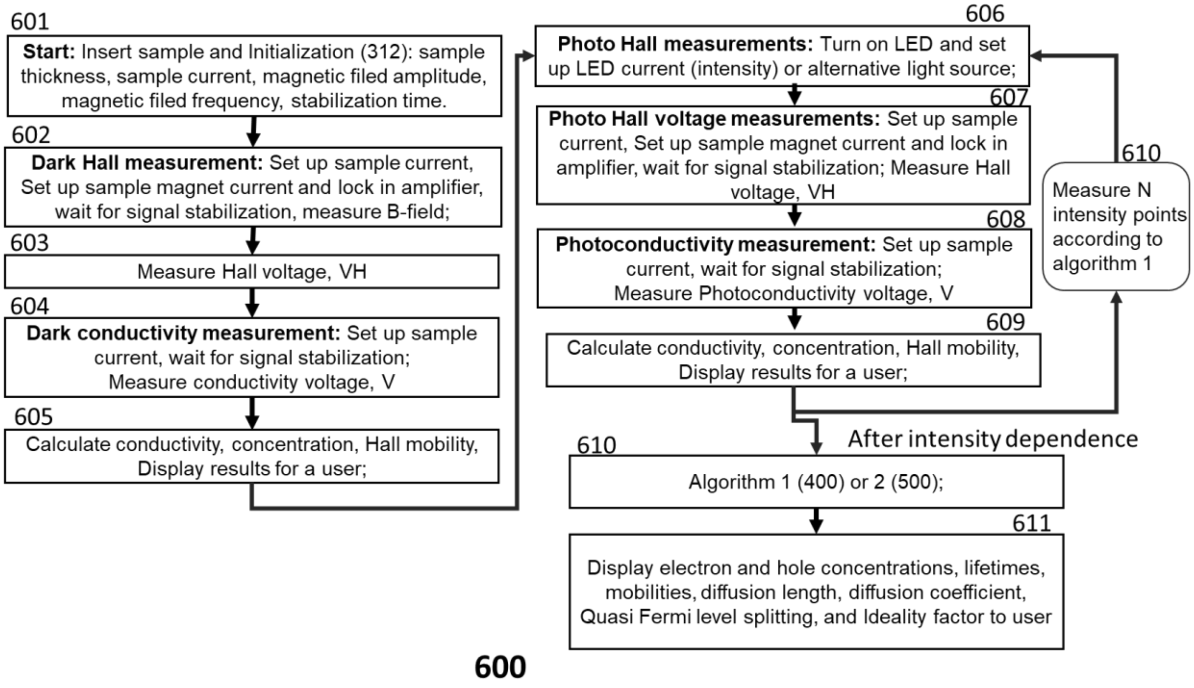

Figure S6| CLIMAT measurements program in AC field regime.

The program (700) for DC magnetic field (300) is shown in **Figure S7**. The programs (600 and 700) can be implemented in any programming language and installed on PC (214), from which controlling the instruments, algorithms, and data is performed.

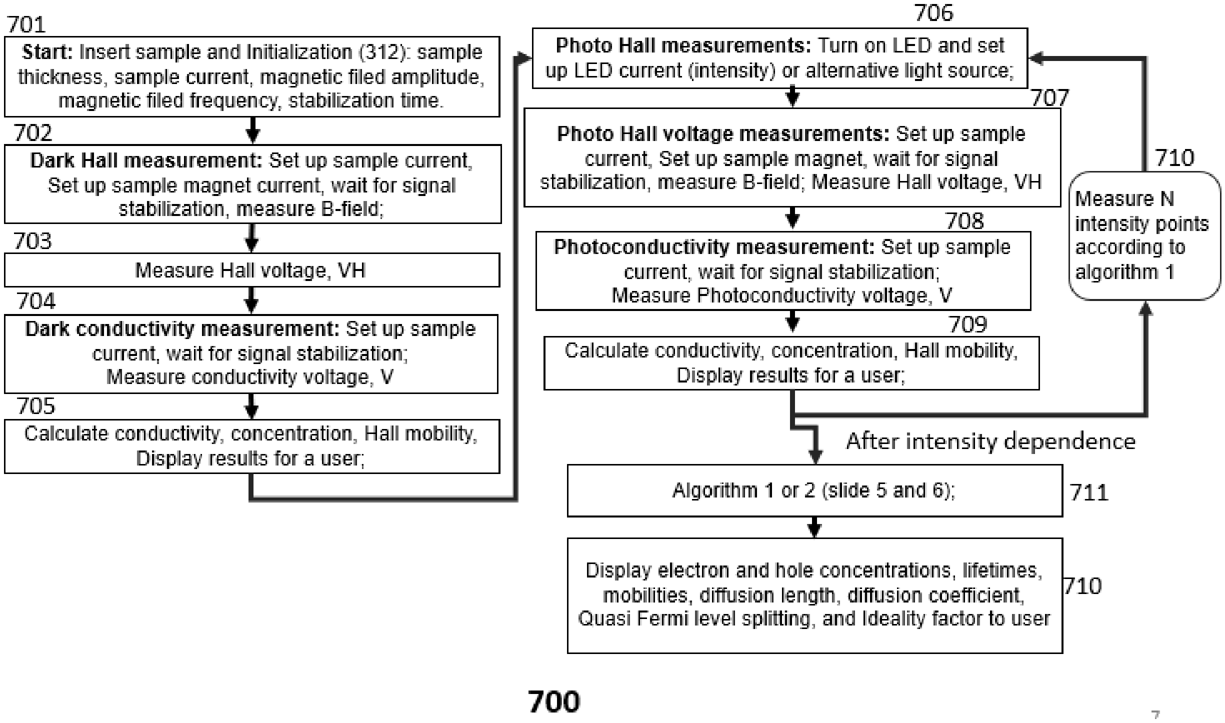

Figure S7| CLIMAT measurements program 2 for DC magnetic field (for (Method B)

1.7 Correction algorithm for mobility in the case of parasitic conductivity

The correction algorithm (900) is given in **Figure S8** to correct the value of mobility and find the correct dark concentration and parasitic conductivity value. As an initial step (901), conductivity (σ) and Hall mobility (μH) as a function of generation rate are used as input data for correction algorithm 1 (900). After correcting mobility (final step 905), algorithm 1 (400) can proceed with step (407) to calculate values of hole and electron mobility.

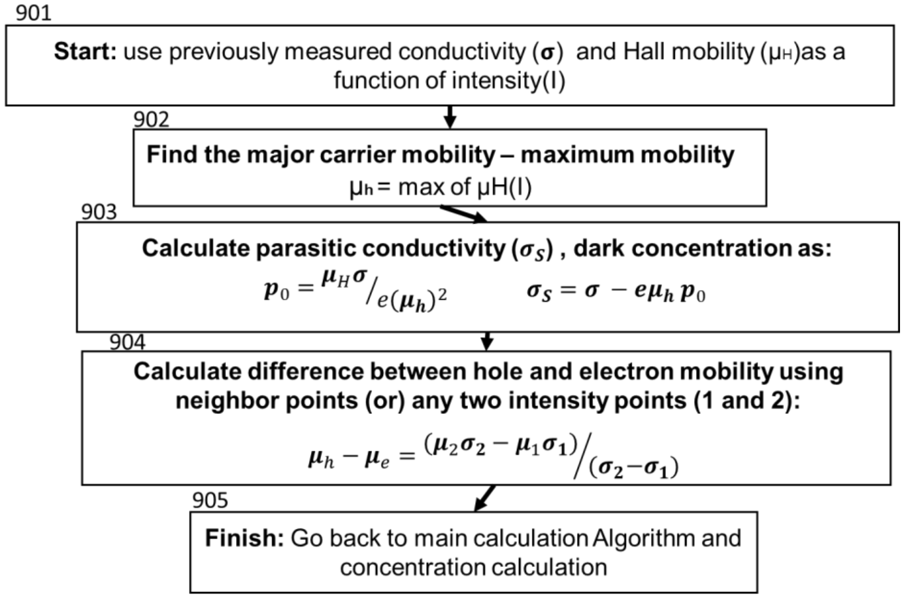

900

Figure S8 | Correction algorithm of the mobility properties in the case of parasitic conductivity or low (decreasing) Hall effect signal

1.8 Correction algorithm for concentration in the case of undetectable Hall effect signal

The correction algorithm (1000) is given in **Figure S9** to correct the value of concentration and finds the correct dark concentration and parasitic conductivity value. In the initial step (1001), conductivity (σ) as a function of generation rate and electron mobility (μ_e) and hole mobility (μ_h) are used as input data for correction algorithm 1 (1000).

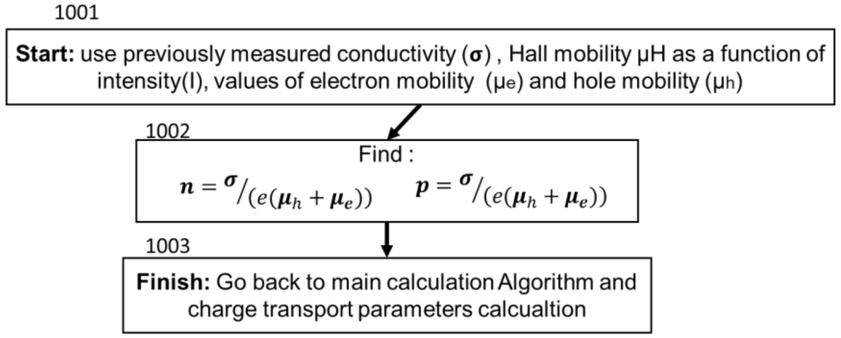

1000

Figure S9 | Correction of the carrier concentration in the case of a low Hall effect signal

2 Charge transport simulations

2.1 Simulation of Hall mobility in semiconductors with and without parasitic conductivity:

Figure S10 demonstrates the simulation of Hall mobility as a function of generation intensity in materials with different mobility and parasitic conductivity values. Firstly, we consider semiconductors with $\mu_e = \mu_h = 1$ cm^{2}V^{-1}s^{-1}. We assume the presence of one deep trap with concentration 10^{14} cm^{-3}, hole capture cross-section 2×10^{-15} cm^2, electron capture cross-section 5×10^{-15} cm^2, and activation energy 0.6 eV to mimic charge transport in a typical semiconductor. The theoretical n and p are calculated using Eq. S4-S6.

The presence of parasitic conductivity decreases the true value of Hall mobility (Figure S10a at low intensities or in the dark). Using illumination, the Hall mobility can be increased up to the value of correct free hole mobility μ_h=1 cm^{2}V^{-1}s^{-1}. If electron and hole mobilities are similar, the Hall mobility will then decrease from the free hole mobility value to the difference between hole and electron mobility (Equation 4).

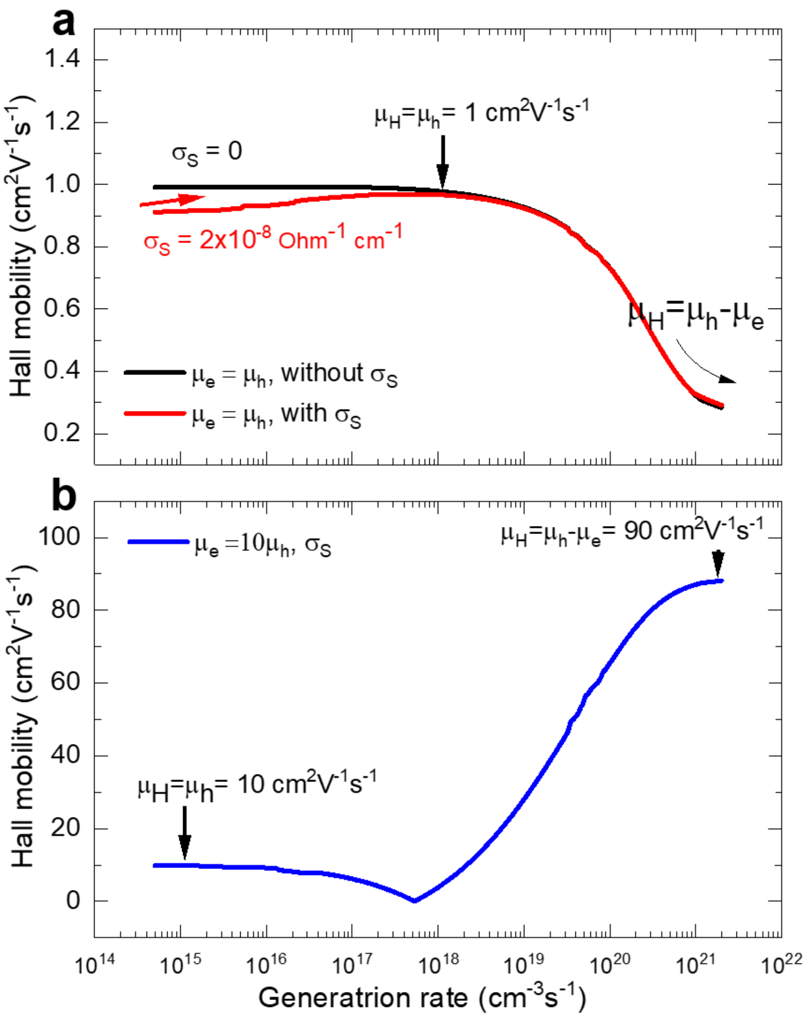

Figure S10 | Hall mobilities in different types of materials. (a) Electron mobility is the same as hole mobility, (b) Electron mobility is much larger than hole mobility.

2.2 Small signal correction and parasitic conductivity:

When electron and hole mobilities are the same or similar, the Hall signal can drop to zero. In such conditions, the correction can be used to find the difference between electron and hole mobility. According to Eq. 5 and conductivity $\sigma = (\sigma_S + e\mu_h p + e\mu_e n)$, the $\sigma\mu_H$ product can be written in the form of Equation S3 assuming similar values of electron and hole photo-exited concentration ($\Delta n \approx \Delta p$):

$$\sigma\mu_H = (\mu_h - \mu_e)(\sigma - \sigma_s - e\mu_h p_0)/e + p_0\mu_h^2 \qquad \text{S3}$$

where p_0 is the majority dark hole concentration. The value of conductivity $\boldsymbol{\sigma}$ is the only parameter which depends on the intensity in Equation S3 on the right side. Thus, we can subtract points measured at two different intensities to find the difference between electron and hole mobility (Equation 5).

$\Delta\mu$ is determined based on respective Hall mobilities $\mu_H(I_C)$ and $\mu_H(I_D)$, and conductivities $\sigma(I_C)$ and $\sigma(I_D)$ acquired at two different illumination intensities I_C and I_D respectively, wherein I_D exceeds I_C by at least 25%, and $I_D > I_C$, particularly from $\Delta\mu = \left|{(\mu_H(I_D)\sigma(I_D) - \mu_H(I_C)\sigma(I_C))}/{(\sigma(I_D) - \sigma(I_C))}\right| = |\beta|$. wherein mobility of minor carriers is determined by adding the major carrier mobility and $\Delta\mu$ if $\beta < 0$ and ($\mu_H(I_D) < \mu_H(I_C)$), or if $\beta > 0$ and $\mu_H(I_D) > \mu_H(I_C)$ wherein mobility of minor carriers is determined by subtracting $\Delta\mu$ from the majority carrier mobility, if $\beta > 0$ and $\mu_H(I_D) < \mu_H(I_C)$.

2.3 Charge recombination simulation:

The following equations were employed for the carrier transport model involving the non-radiative [9], and radiative recombination channels:

$$0 = \frac{dn}{dt} = G - C_{bb}(np - n_i^2) - n\sigma_e v_e(N_t - n_t) \qquad \text{S4}$$

$$0 = \frac{dn_t}{dt} = G - n\sigma_e v_e(N_t - n_t) - p\sigma_h v_h n_t \qquad \text{S5}$$

$$0 = \frac{dp}{dt} = G - C_{bb}(np - n_i^2) - p\sigma_h v_h n_t \qquad \text{S6}$$

Where G is generation rate; C_{bb} is radiative constant; n_t and $\sigma_{e/h}$ are trap density and capture cross-section, n_0 and p_0 are dark carrier concentrations. The initial guess values of capture cross-section and activation energies for a fit of experimental CLIMAT data by the least square method were adapted from one of our previous study[10]. Note that knowledge of n and p as a function of intensity allows fitting unambiguously to capture cross-section and concentration of trap due to the unique balance between n and p. The demonstration of the effect on parameter variation on the fit quality is shown in **Figure S11**. The simultaneous increase in trap concentration and decrease in capture cross-section fail to fit the experimental data. This demonstrates that CLIMAT data consists of a unique fingerprint of trap occupation (*p(G)-n(G)*), from which trap parameters can be reliably determined.

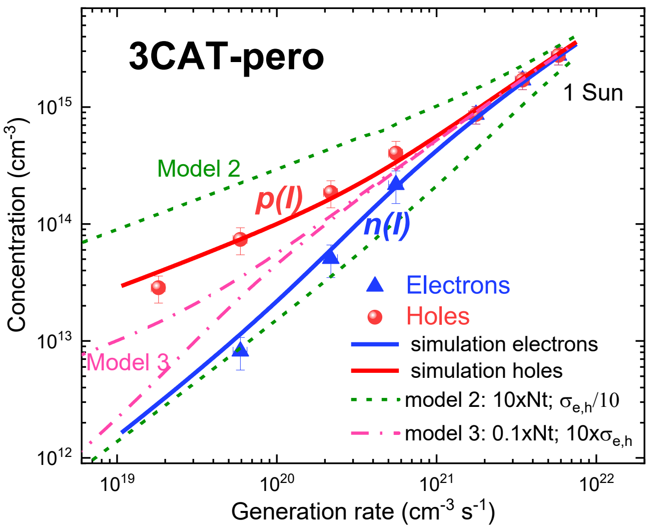

Figure S11 | Variation of fit parameters for halide perovskite CLIMAT data.

Considering Eq. S4-6 , the lifetime of electrons and holes is shown in Eq. S7-8. It includes contributions that depend on the generation rate, encompassing both radiative and non-radiative (trap-associated) recombination processes and considers trap filling and occupation saturation.

$$\tau_e = \frac{\Delta n}{G} = \frac{1}{C_{bb}p + \sigma_e v_e (N_t - n_t)} \qquad \text{S7}$$

$$\tau_h = \frac{\Delta p}{G} = \frac{1}{C_{bb}n + \sigma_h v_h n_t} \qquad \text{S8}$$

Auger recombination is neglected, as it only plays a role in carrier recombination at concentrations exceeding[75] 10^{18} cm^{-3}, which is three orders of magnitude higher than the concentrations observed in our study.

3. Validation of CLIMAT results by THz, TRMC, TRPL, QFLS, and ToF characterisation

To validate the charge transport properties probed by CLIMAT, we conducted a comprehensive set of additional measurements using alternative characterization techniques. First, we validate lifetime in Si and perovskite, Fig. S12a-b. The electron drift mobility in silicon is validated by THz spectroscopy, Fig. S12c.

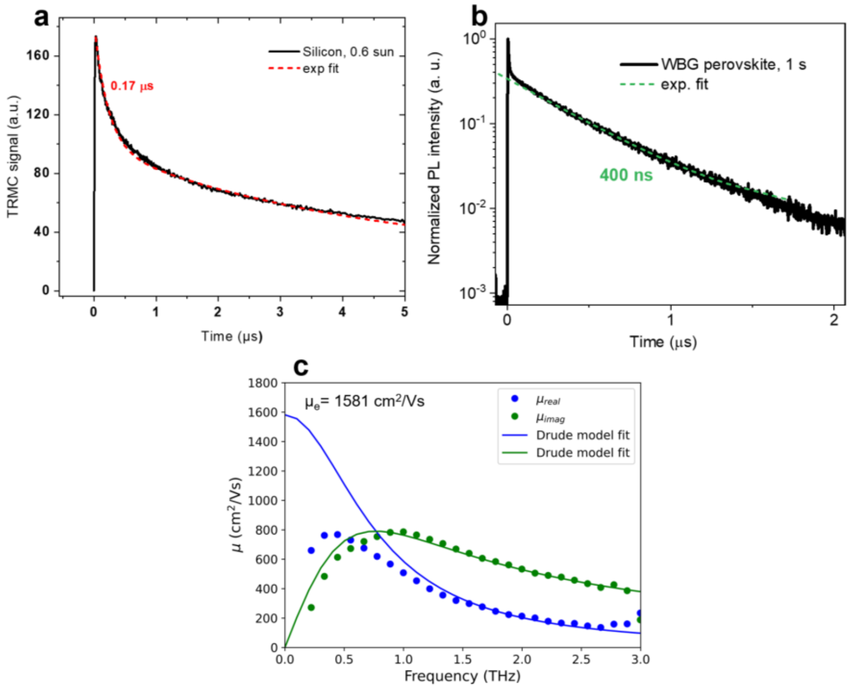

Figure S12 | Lifetime in (a) silicon and (b) halide perovskite by TRMC and TRPL measured at 0.6 and 1 sun equivalent. (c) THz measurements of mobility in silicon sample.

Time-of-flight (ToF) is considered one of the most precise techniques for detecting charge transport properties of carriers[72]. This precision arises from its direct observation of signals generated by carriers, particularly in the transit time, which allows for the direct observation of the moment when carriers reach another electrode. However, it's worth noting that ToF measurements are primarily conducted on single crystal samples (SC) to achieve clear transit time regions.

To validate the results obtained through the CLIMAT method, we have undertaken a comparative analysis of ToF-derived lifetime and mobility in systems where both ToF and CLIMAT measurements are applicable. Specifically, we have focused on single crystal samples of $MAPbBr_3$ (sc-$MaPbBr_3$) and $CsPbBr_3$ (sc-$CsPbBr_3$), as shown in Figure S13 and Table S3. CLIMAT and Time-of-Flight (ToF) measurements exhibit a high level of agreement in terms of both carrier lifetime and mobility. This substantial agreement underscores the reliability and consistency of the results obtained through both techniques, further validating the accuracy of our findings.

Table S3. Comparison of lifetime and mobility found by ToF and CLIMAT at low injection regime in single crystal samples (sc)

	**sc-$CsPbBr_3$**	**sc-$MAPbBr_3$**
**Lifetime (μs), CLIMAT**	1300	200
**Lifetime (μs), ToF**	1000	103
**Mobility($cm^2V^{-1}s^{-1}$), CLIMAT**	26	11
**Mobility ($cm^2V^{-1}s^{-1}$), ToF**	23	12.3

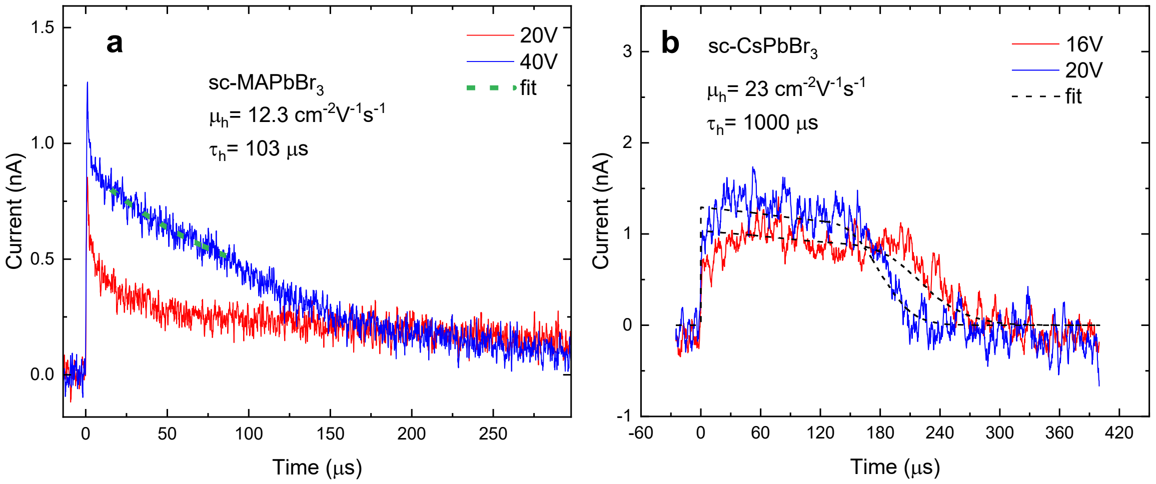

.

Figure S13 | ToF measured in (a) sc-MaPbBr$_3$ and (b) sc-CsPbBr$_3$ and at low intensity ($p<10^{13}$ cm^{-3}).

Figure S14 illustrates PLQY measurements that validate the accuracy of the Quasi-Fermi Level Splitting (QFLS) values determined by CLIMAT in both silicon and thin film perovskite samples.

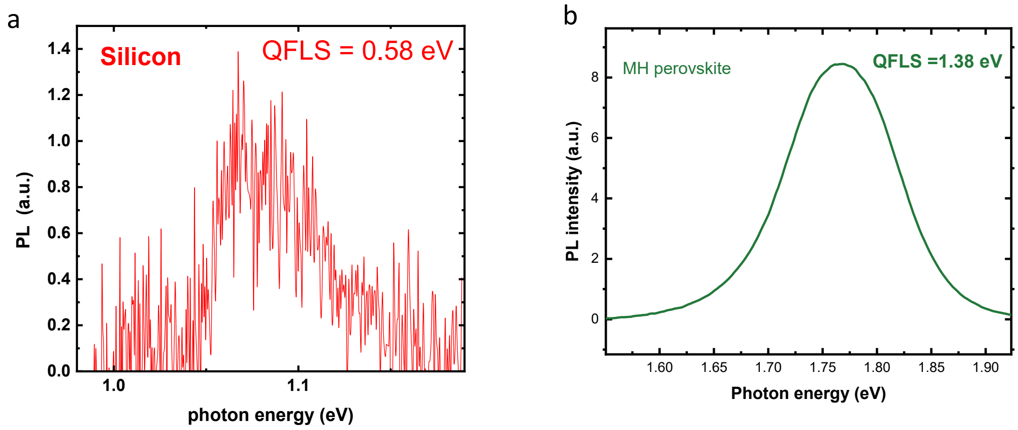

Figure S14 | PLQY measured in silicon (a) and perovskite (b) samples

4. Stability of CLIMAT signal and transport properties in perovskite during the measurement time

To illustrate the stability of the CLIMAT signal and transport properties of the examined perovskite sample, we depict the core transport parameters over time at the highest illumination intensity of 1 sun utilized in this study (see Figure S15). As the study was perturbed by light

illumination, the semiconductor sample required time to attain steady-state conditions. Once a steady state was achieved, the sample's transport properties remained stable, exhibiting a standard deviation of 5% attributable to noise in materials. Similar behavior was previously observed by us in several material systems [45,76]. Note also that the intensity dependence of CLIMAT concentration is in perfect agreement with the theoretical simulation of intensity dependance Figure 4d.

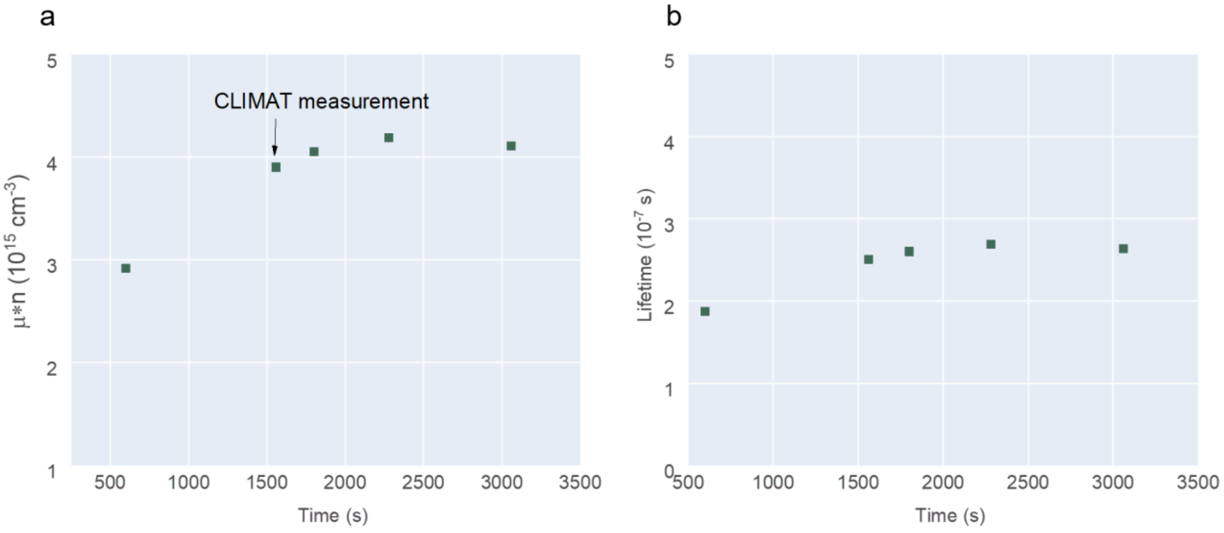

Figure S15| CLIMAT measurements in perovskite sample a) μ×n product (b) lifetime of free carriers
