# Supplementary material for: Resolving electron and hole transport properties in semiconductor materials by constant light-induced magneto transport
Source: Nat Commun. 2024 Jan 5;15:316. doi: 10.1038/s41467-023-44418-1 (PMC10770130; doi:10.1038/s41467-023-44418-1)
Supplement: Supplementary file 3 — Reporting Summary [file 41467_2023_44418_MOESM3_ESM.pdf]

nature research

Corresponding author(s): Artem Musiienko

Lasing Reporting Summary

Nature Research wishes to improve the reproducibility of the work that we publish. This form is intended for publication with all accepted papers reporting claims of lasing and provides structure for consistency and transparency in reporting. Some list items might not apply to an individual manuscript, but all fields must be completed for clarity.

For further information on Nature Research policies, including our data availability policy, see Authors & Referees.

• Experimental design

Please check: are the following details reported in the manuscript?

1. Threshold

Plots of device output power versus pump power over a wide range of values indicating a clear threshold

- [ ] Yes
- [x] No

We do not have device output power versus pump power over a wide range of values indicating a clear threshold. This paper is not relevant for laser systems and does not report any related quantities

2. Linewidth narrowing

Plots of spectral power density for the emission at pump powers below, around, and above the lasing threshold, indicating a clear linewidth narrowing at threshold

- [ ] Yes
- [x] No

We do not have linewidth narrowing

Resolution of the spectrometer used to make spectral measurements

- [ ] Yes
- [ ] No

State where this information can be found in the text.

Explain why this information is not reported/not relevant.

3. Coherent emission

Measurements of the coherence and/or polarization of the emission

- [ ] Yes
- [x] No

We do not have coherent emission

4. Beam spatial profile

Image and/or measurement of the spatial shape and profile of the emission, showing a well-defined beam above threshold

- [ ] Yes
- [x] No

We do not have beam spatial profile

5. Operating conditions

Description of the laser and pumping conditions
Continuous-wave, pulsed, temperature of operation

- [ ] Yes
- [x] No

We do not have pumping condition

Threshold values provided as density values (e.g. W cm^{-2} or J cm^{-2}) taking into account the area of the device

- [ ] Yes
- [x] No

We do not have threshold values

6. Alternative explanations

Reasoning as to why alternative explanations have been ruled out as responsible for the emission characteristics
e.g. amplified spontaneous, directional scattering; modification of fluorescence spectrum by the cavity

- [ ] Yes
- [x] No

This paper is not relevant for laser systems and does not report any related quantities

7. Theoretical analysis

Theoretical analysis that ensures that the experimental values measured are realistic and reasonable
e.g. laser threshold, linewidth, cavity gain-loss, efficiency

- [ ] Yes
- [x] No

This paper is not relevant for laser systems and does not report any related quantities

8. Statistics

Number of devices fabricated and tested

- [x] Yes
- [ ] No

3

Statistical analysis of the device performance and lifetime (time to failure)

- [ ] Yes
- [x] No

This paper is not relevant for laser systems and does not report any related quantities

March 2018
